# Increased frequency of β cells with abnormal NKX6.1 expression in type 2 diabetes but not in subjects with higher risk for type 2 diabetes

**DOI:** 10.1186/s12902-021-00708-7

**Published:** 2021-03-12

**Authors:** Tengli Liu, Peng Sun, Jiaqi Zou, Le Wang, Guanqiao Wang, Na Liu, Yaojuan Liu, Xuejie Ding, Boya Zhang, Rui Liang, Shusen Wang, Zhongyang Shen

**Affiliations:** 1 Organ Transplant Center, Tianjin First Central Hospital, Nankai University, Building A, Fukang Road 24#, Nankai area, Tianjin City, 300192 China; 2 NHC Key Laboratory for Critical Care Medicine, Tianjin First Central Hospital, Tianjin, 300384 China; 3 Tianjin Key Laboratory for Organ Transplantation, Tianjin First Central Hospital, Tianjin, 300192 China

**Keywords:** NKX6.1, Age, Obesity, HbA1c, β-Cell dedifferentiation

## Abstract

**Background:**

NKX6.1 is a transcription factor for insulin, as well as a marker for β cell maturity. Abnormal NKX6.1 expression in β cells, such as translocation from the nucleus to cytoplasm or lost expression, has been shown as a marker for β cell dedifferentiation.

**Methods:**

We obtained pancreatic sections from organ donors and immunofluorescence staining with NKX6.1 and insulin was performed to characterize NKX6.1 expression in subjects with or without type 2 diabetes mellitus (T2DM).

**Results:**

Our results showed that cells with insulin expression but no nucleic NKX6.1 expression (NKX6.1^Nuc-^Ins^+^), and cells with cytoplasmic NKX6.1 expression but no insulin expression (NKX6.1^cyt^Ins^−^) were significantly increased in T2DM subjects and positively correlated with glycated hemoglobin (HbA1c), indicating the elevated β cell dedifferentiation with NKX6.1 inactivation in T2DM. To investigate whether β cell dedifferentiation has initiated in subjects with higher risks for T2DM, we next analyzed the association between β-cell dedifferentiation level in ND subjects with different ages, body mass index, and HbA1c. The results showed the absolute number and percentage of dedifferentiated β cells with NKX6.1 inactivation did not significantly change in subjects with advanced aging, obesity, or modest hyperglycemia, indicating that the β cell dedifferentiation might mainly occur after T2DM was diagnosed.

**Conclusion:**

Our results suggested that NKX6.1 expression in β cells was changed in type 2 diabetic subjects, evidenced by significantly increased NKX6.1^Nuc-^Ins^+^ and NKX6.1^cyt^Ins^−^ cells. This abnormality did not occur more frequently in subjects with a higher risk for T2DM, suggesting that β cell dedifferentiation might be secondary to the pathological changes in T2DM.

**Supplementary Information:**

The online version contains supplementary material available at 10.1186/s12902-021-00708-7.

## Background

463 million adults are currently affected by diabetes worldwide in 2019, around 90% of which are type 2 diabetes mellitus (T2DM) [1]. There are 116 million diabetic patients in China, being the largest in the world [1, 2]. β-cell deficit is one of the root causes in the development of type 2 diabetes [3, 4], and our previous study showed a significant decrease in β-cell mass in Chinese patients with type 2 diabetes [5]. One of the main causes of β-cell deficit in type 2 diabetes is the loss of key transcription factors in β cells, which makes β cells back to a “dedifferentiated” state, including the homeobox protein NKX6.1 [6–8]. NKX6.1 is one of the key transcription factors that are engaged in early and late pancreatic β cell specification [9]. The gene regulatory network controlled by transcription factor NKX6.1 is also necessary to maintain the function and identity of mature β cells [10, 11], and the overexpression of Nkx6.1 in adult mouse β cells of mice resulted in elevated glucose stimulated insulin secretion (GSIS) [10], yet the inactivation of Nkx6.1 could lead to a decrease in insulin secretion, causing diabetes [11]. Meanwhile, Nkx6.1 inactivation in adult mouse β cells could [11] induce the expression of progenitor cell markers [6, 7, 11–13], which exhibited a dedifferentiation stage. These results are consistent with the findings that NKX6.1 expression is decreased in β cells from obese-diabetic mice and human T2DM islets compared with that in non-diabetic ones [7, 14]. Therefore, NKX6.1 inactivation has been used as a protein marker for β cell dedifferentiation.

It has been reported that using NKX6.1 staining can discriminate a subset of dedifferentiated β cells, and the NKX6.1 defined β cell dedifferentiation level is elevated in T2DM patients or T2DM animal models. However, whether β cell dedifferentiation has higher level in subjects with higher risks for diabetes compared with those with lower risks is unknown. The present study hypothesized that β cell dedifferentiation level is higher in the former than in the latter. To test our hypothesis, we evaluated the β cell dedifferentiation level in the pancreases of organ donors with differential risk for diabetes, such as different age layers, BMI, and HbA1c [15–18], as well as in T2DM patients, using NKX6.1 as dedifferentiation marker.

## Methods

### Human subjects

Human pancreata from 60 organ donors were obtained with between December 2016 and December 2019 with informed consents for research. We only included subjects who underwent a full autopsy within 4 h of death, pancreatitis, pancreatic surgery or cancer, and showed no signs of pancreatic autolysis. The information of organ donors was shown in Tables 1 and 2. Organ donors were firstly classified as ND with HbA1c (< 6.4%) and no history of diabetes (*n* = 40) and BMI, and sex matched T2D with HbA1c (≥ 6.5%) or clinical history of diabetes (T2D, *n* = 20). The ND controls were further sub-divided into different groups and the average data of each group was shown in Table 2. ND subjects were divided into four age groups (30–49, 40–49, 50–59, 60–79 years) matched by BMI, HbA1c, sex and four BMI groups (< 23, 23–25, 25–30, ≥ 30) matched by age, HbA1c, and sex. Besides the ND subjects were divided into HbA1c < 5.7% and HbA1c 5.7–6.4% which matched by age, BMI, sex. The Medical Ethical Committee of Tianjin First Central Hospital has approved all procedures (Review number:2016N079KY).

**Table 1:** Organ donor information from ND (Non-diabetic) and T2DM (type 2 diabetes mellitus) subjects

	BMI (kg/m2)	Age (years)	HbA1c (%)	Sex (males/females)
ND (*n* = 40)	26.27 ± 0.75	48.33 ± 1.38	5.34 ± 0.07	32/8
T2DM (*n* = 20)	25.72 ± 0.62	53.60 ± 1.89^*^	7.58 ± 0.28^†^	16/4

Data are shown as mean ± SEM. ^*^compared with ND, *P* < 0.05; ^†^compared with ND, *P* < 0.0001

**Table 2:** Organ donor information from ND (Non-diabetic) subjects

	N	BMI (kg/m2)	Age (years)	HbA1c (%)	sex (M/F)
HbA1c (%)					
HbA1c (%, < 5.7)	32	25.69 ± 0.77	47.66 ± 1.60		27/5
HbA1c (%, 5.7–6.4)	8	28.59 ± 2.09	51.00 ± 2.54		5/3
Age (years)					
30–39	5	28.34 ± 3.73		5.38 ± 0.15	4/1
40–49	18	25.51 ± 0.85		5.24 ± 0.10	16/2
50–59	13	27.06 ± 1.45		5.46 ± 0.11	9/4
60–79	4	24.53 ± 0.61		5.30 ± 0.32	3/1
BMI (kg/m2)					
< 23	11		48.00 ± 2.09	5.35 ± 0.15	9/2
23–25	7		47.86 ± 3.49	5.40 ± 0.16	7/0
25–30	16		48.94 ± 2.57	5.24 ± 0.11	13/3
≥ 30	6		47.83 ± 3.51	5.52 ± 0.12	3/3

*M/F* Males/Females. Data are shown as mean ± SEM

### Immunofluorescence analysis

Human Pancreatic tissue was fixed with 10% formalin, followed by dehydration, paraffin embedding, and sectioning at 3 μm. Sections were then stained according to the protocol in one of our earlier studies [5]. After antigen retrieval of In brief, after deparaffinization, the paraffin sections were blocked with blocking buffer (10% FBS, 0.1% Triton X-100) for 30 min at 37°Cfollowed by sequential incubations with anti-NKX6.1 (1:500, NBP1–49672, Novus, USA) and anti-insulin (1:200, ab7842, Abcam, USA) at 4 °C overnight and secondary antibodies (Alexa Fluor 488 AffiniPure Goat Anti-Guinea Pig IgG H&L, 1:200, 106–545-003, Jackson Immunoresearch Laboratories and Molecular Probes, USA; Rhodamine (TRITC) AffiniPure Goat Anti-Rabbit IgG H&L, 1:100, 111–025-003, Jackson Immunoresearch Laboratories and Molecular Probes, USA) for 30 min at 37 °C, and then counterstained with DAPI. Pancreatic tissues were scanned by Pannoramic MIDI and Pannoramic Viewer (3DHistech).

Quantifications were performed in a blinded fashion using the CytoNuclear count function of the Image Pro Plus software (Media Cybernetics, Silver Spring, Maryland). Cells with a strong nucleic NKX6.1 immunreactivity in islet were described as nucleus NKX6.1^+^ cells, and cells with cytoplasmic or no NKX6.1 immunreactivity were described as nucleus NKX6.1^−^ cells.

### Statistical analysis

Figure drawing and data processing were performed using GraphPad Prism v7.0 (GraphPad Software, La Jolla, CA, USA). Quantitative data were shown as means ± SEM. Student’s t test was used for analyzing the group differences. The sex-ratio were analyzed by chi-square test. *P* < 0.05 were considered statistically significant.

## Results

### The characteristics of NKX6.1 defined β-cell dedifferentiation

**Fig. 1:**
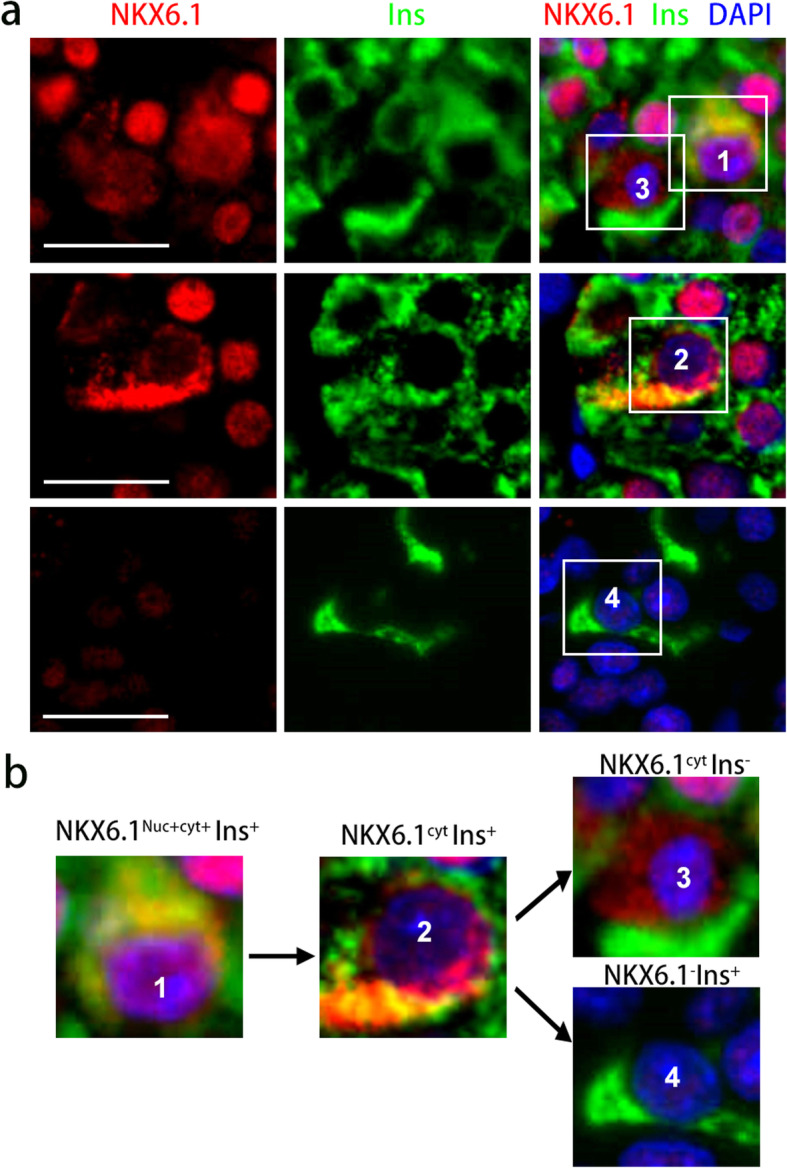
Characteristics of dedifferentiated β cells identified by NKX6.1. **a** Representative immunofluorescence images of pancreatic sections with NKX6.1 and Insulin (Ins). **b** Relationships among different dedifferentiated β cell labeled by white boxes in (**a**). Scale bars: 20 μm, Red: NKX6.1, Green: Ins, Blue: DAPI

**Fig. 2:**
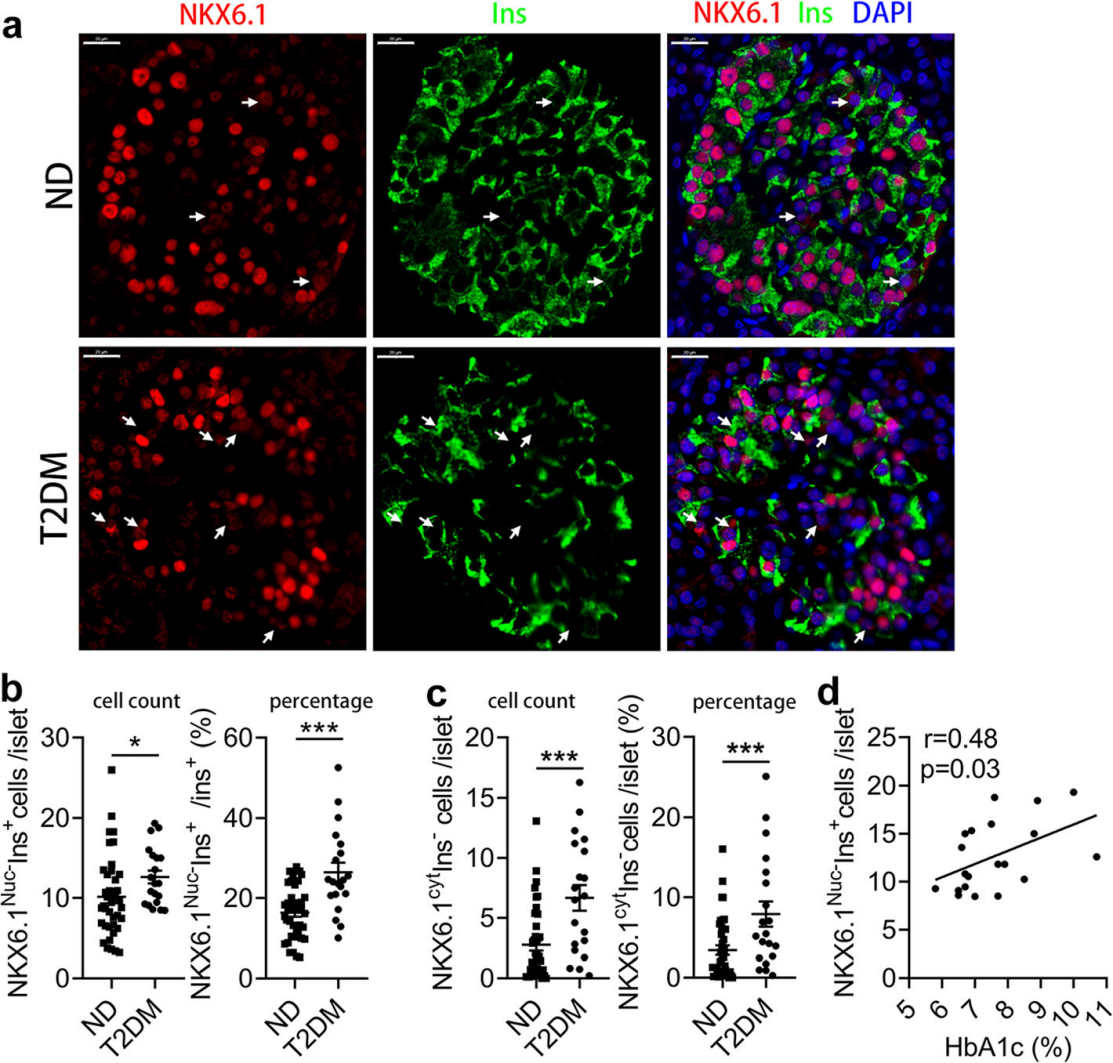
Representative immunofluorescence images of pancreatic sections of non-diabetic (ND) subjects and T2DM subjects. **a** Representative images of immunostaining with Insulin and NKX6.1 in the pancreatic sections of non-diabetic (ND) subjects and T2DM subjects. White arrows marked dedifferentiated β cells with NKX6.1 dislocation. Scale bar: 20 μm, Red: NKX6.1, Green: Ins, Blue: DAPI. **b** Quantification of NKX6.1^Nuc-^Ins^+^ cell count and percentage in β cells. **c** Quantification of NKX6.1^cyt^Ins^−^ cell count and percentage per islet. **d** Correlation between NKX6.1^Nuc-^Ins^+^ cells per islet with HbA1c in T2DM subjects (Simple linear regression). *n* = 40 for non-diabetic (ND) subjects; *n* = 20 for T2DM subjects. Data were shown as mean ± SEM. *P < 0.05. ***P < 0.001

By immunofluorescence staining with insulin and NKX 6.1 in human pancreatic tissue sections, we identified two types of dedifferentiated β cells with NKX6.1 inactivation: 1) Cells that are insulin-positive but without nucleic NKX6.1 expression are at the early stage of dedifferentiated β cells (NKX6.1^Nuc-^Ins^+^, including β cells with cytoplasmic NKX6.1 expression or no NKX6.1 expression, Fig. 1a-b); 2) Cells that are insulin-negative but with cytoplasmic expression of NKX6.1 are at the late stage of dedifferentiated β cells (NKX6.1^cyt^Ins^−^, Fig. 1a-b). The mean absolute number of NKX6.1^Nuc-^Ins^+^ cells per islet was 2.69 times that of NKX6.1^cyt^Ins^−^ cells. In addition, we also captured the β cells with the expression of NKX6.1 in both nucleus and cytoplasm in the earlier stage of dedifferentiated β cells, but that was rare. NKX6.1 dislocation or lost expression were signs of NKX6.1 inactivation. These results suggest different stages of NKX6.1 inactivation in the dynamic process of β-cell dedifferentiation.

### The level of β cell dedifferentiation with NKX6.1 inactivation was elevated and positively correlated with HbA1c in type 2 diabetic subjects

We next evaluated the level of β-cell dedifferentiation with NKX6.1 inactivation in ND and T2DM subjects (Fig. 2a). The absolute number of NKX6.1^Nuc-^Ins^+^ cells per islet in T2DM subjects were increased by 24.41% (Fig. 2b, *P* < 0.05), and the percentage in β cells increased by 61.56%, compared with ND subjects (Fig. 2b, *P* < 0.001). The absolute number and percentage of NKX6.1^cyt^Ins^−^ cells per islet in T2D subjects increased by 2.41 and 2.31 fold, respectively (Fig. 2c, *P* < 0.001, *P* < 0.001). We also found NKX6.1^Nuc-^Ins^+^ cells have a significant correlation with HbA1c in T2DM subjects (Fig. 2d, *P = 0.03*). These results suggest that the level of β-cell dedifferentiation in T2DM islet is elevated and aggravated by hyperglycemia.

### Modest hyperglycemia, advanced age, or obesity did not affect the level of β cell dedifferentiation with NKX6.1 inactivation

To evaluate the effect of modest hyperglycemia on β-cell dedifferentiation with NKX6.1 inactivation, the non-diabetic samples were further divided into the non-prediabetic group with normal glycaemia (HbA1c < 5.7%) and the prediabetic group with modest hyperglycemia (HbA1c: 5.7–6.4%). The absolute number of NKX6.1^Nuc-^Ins^+^ cells per islet was 10.45 ± 0.91 in non-prediabetes and 9.00 ± 1.67 in prediabetes, respectively, and the percentage in β cells was 16.31 ± 1.15% and 16.68 ± 2.40% (Table 3). The absolute number of NKX6.1^cyt^Ins^−^ cells per islet was 2.95 ± 0.55 and 2.04 ± 0.91, respectively, and the percentage per islet was 3.57 ± 0.69% and 2.84 ± 1.15%, respectively (Table 3).

**Table 3:** Effect of high risk factors for type 2 diabetes mellitus on NXK6.1 Ins^+^ cells and NKX6.1cyt Ins + cells per islet in pancreas

	NXK6.1^Nuc-^Ins^+^ cells /islet	NXK6.1^Nuc-^Ins^+^ /ins^+^ (%)	NXK6.1^cyt^Ins^−^ cells /islet	NXK6.1^cyt^Ins^−^ cells /islet (%)
HbA1c (%)				
< 5.7%	10.45 ± 0.91	16.31 ± 1.15	2.95 ± 0.55	3.57 ± 0.69
5.7–6.4%	9.00 ± 1.67	16.68 ± 2.40	2.04 ± 0.91	2.84 ± 1.15
Age (years)				
30–39	9.32 ± 4.20	13.19 ± 3.56	3.05 ± 1.16	3.66 ± 1.38
40–49	11.63 ± 0.92	18.20 ± 1.16	2.71 ± 0.82	3.24 ± 1.00
50–59	8.95 ± 1.29	15.72 ± 2.02	2.16 ± 0.58	3.00 ± 0.81
60–79	8.57 ± 2.07	14.40 ± 4.03	4.67 ± 2.07	5.34 ± 2.64
BMI (kg/m2)				
< 23	12.64 ± 1.64	18.46 ± 1.35	3.10 ± 0.87	3.80 ± 1.15
23–25	9.69 ± 1.71	15.90 ± 2.21	3.65 ± 1.81	4.39 ± 2.21
25–30	9.41 ± 1.24	15.92 ± 1.96	2.45 ± 0.69	3.05 ± 0.85
≥ 30	8.18 ± 1.85	14.40 ± 2.73	1.98 ± 0.53	2.61 ± 0.67

Data are shown as mean ± SEM. No significant difference was observed between different groups

To analyze the effects of aging on β-Cell dedifferentiation with NKX6.1 inactivation, we further divided non-diabetic subjects into four groups with ages of 30–39, 40–49, 50–59, and 60–79, respectively. The absolute number of NKX6.1^Nuc-^Ins^+^ cells per islet was 9.32 ± 4.20, 11.63 ± 0.92, 8.95 ± 1.29, and 8.57 ± 2.07 in the four groups respectively, the percentages in β cells was 13.19 ± 3.56%, 18.20 ± 1.16%, 15.72 ± 2.02%, and 14.40 ± 4.03%, respectively (Table 3). The absolute number of NKX6.1^cyt^Ins^−^ cells per islet was 3.05 ± 1.16, 2.71 ± 0.82, 2.16 ± 0.58, and 4.67 ± 2.07, the percentage per islet was 3.66 ± 1.38%, 3.24 ± 1.00%, 3.00 ± 0.81%, and 5.34 ± 2.64%, respectively (Table 3).

To analyze the effects of obesity on β-cell dedifferentiation with NKX6.1 inactivation, non-diabetic samples were divided into four groups according to the BMI standards for Asians special BMI: < 23 (normal), 23–25 (overweight), 25–30 (obesity), ≥30 (over-obesity). The absolute number of NKX6.1^Nuc-^Ins^+^ cells per islet was 12.64 ± 1.64, 9.69 ± 1.71, 9.41 ± 1.24, and 8.18 ± 1.85, respectively, the percentage in β cells was 18.46 ± 1.35%, 15.90 ± 2.21%, 15.92 ± 1.96%, and 14.40 ± 2.73%, respectively (Table 3). The absolute number of NKX6.1^cyt^Ins^−^ cells per islet was 3.10 ± 0.87, 3.65 ± 1.81, 2.45 ± 0.69, and 1.98 ± 0.53, respectively, and the percentage per islet was 3.80 ± 1.15%, 4.39 ± 2.21%, 3.05 ± 0.85%, and 2.61 ± 0.67%, respectively (Table 3).

By statistical analysis, we found that there was no association between the number and percentage of NKX6.1^Nuc-^Ins^+^ and NKX6.1^cyt^Ins^−^ cells and modest hyperglycemia, advanced age, and obesity. Further correlation analysis between NKX6.1^Nuc-^Ins^+^ or NKX6.1^cyt^Ins^−^ versus age/BMI/HbA1c found no significant correlation (Additional file 1: Supplementary Fig. 1–3). These results suggested that higher risk factors for type 2 diabetes did not affect β-cell dedifferentiation in the non-diabetic subjects.

## Discussion

The typical expression pattern of NKX6.1 in normal islets was in the nuclei of β cells [6, 7, 19]. In this study, using immunofluorescence staining with NKX6.1 and insulin in human pancreatic sections, we firstly described the overall and subcellular expression pattern of NKX6.1 in human β cells. Here we reported three more types of NKX6.1 expression patterns except its classic nucleic location: (1) both cytoplasmic location and nucleic location in single β cells, (2) only cytoplasmic location, and (3) no expression in β cells. Given NKX6.1 is an transcription factor, its cytoplasmic expression should be with no transcription activity, namely NKX6.1 inactivation. It was a pity that we could not provide the dynamic change of NKX6.1 expression pattern.

In this study, we investigated the expression pattern of NKX6.1 in islets from normal islets to T2DM islets, as well as in subjects with higher risks for developing diabetes. The number or percentage of β cells with abnormal NKX6.1 expression significantly increased in subjects with T2DM. This finding is consistent with previous reports in other studies [7], and supports the concept that β cell dedifferentiation is a mechanism for β cell dysfunction in T2DM.

Our results revealed that the level of β-cell dedifferentiation with NKX6.1 inactivation positively correlated with HbA1c in T2DM subjects but not ND. This result is in accordance with previous report that β cell dedifferentiation was elevated in T2DM islets [7, 14]. However, whether β cell dedifferentiation level has been elevated before T2DM is developed was previously unknown. In this study, we analyzed the level of NKX6.1 inactivation in β cells of subjects with differential age, BMI, or HbA1c. We found no significant increase in NKX6.1-marked β cell dedifferentiation in subjects along with aging, obesity, or the modest increase of glycemia. These results suggested these risk factors didn’t cause significant increase of β cell dedifferentiation level in non-diabetic individuals. It’s possible that β cell dedifferentiation in T2DM subjects would deteriorate with the progression of this disease, but the β cell dedifferentiation is only maintained at a basal level in ND subjects.

## Conclusions

In summary, we report here that the level of β-cell dedifferentiation with NKX6.1 inactivation is significantly increased in Chinese people with type 2 diabetes. Importantly, the three risk factors for type 2 diabetes, including aging, obesity and modest hyperglycemia, did not affect the level of β-cell dedifferentiation in non-diabetic subjects.

## Supplementary Information

**Additional file 1: Supplementary Fig. 1.** Correlations between NKX6.1 inactivation level in β cells and Age in non-diabetic subjects. **a** Correlation between NKX6.1 ^Nuc-^Ins^+^ cells count and percentage with Aging in Non-diabetic subjects (*n* = 40). **b** Correlation between NKX6.1^cyt^Ins^−^ cell count and percentage with Aging in non-diabetic subjects (*n* = 40). **Supplementary Fig. 2.** Correlations between NKX6.1 inactivation level in β cells and BMI in non-diabetic subjects. **a** Correlation between NKX6.1 ^Nuc-^Ins^+^ cells count and percentage with BMI in Non-diabetic subjects (*n* = 40). **b** Correlation between NKX6.1^cyt^Ins^−^ cell count and percentage with BMI in non-diabetic subjects (*n* = 40). **Supplementary Fig. 3.** Correlations between NKX6.1 inactivation level in β cells and HbA1c in non-diabetic subjects. **a** Correlation between NKX6.1 ^Nuc-^Ins^+^ cells count and percentage with HbA1c in non-diabetic subjects (*n* = 40). **b** Correlation between NKX6.1^cyt^Ins^−^ cell count and percentage with HbA1c in non-diabetic subjects (*n* = 40)

## Data Availability

The datasets generated and/or analysed during the current study are not publicly available due to that thourough analyses are still undergoing on the data produced by this project but are available from the corresponding author on reasonable request.

## Abbreviations

T2DM: Type 2 diabetes mellitus
ND: Non-diabetes
HbA1c: Glycated hemoglobin
