# Supplementary material for: Increased frequency of β cells with abnormal NKX6.1 expression in type 2 diabetes but not in subjects with higher risk for type 2 diabetes
Source: BMC Endocr Disord. 2021 Mar 12;21:47. doi: 10.1186/s12902-021-00708-7 (PMC7955633; doi:10.1186/s12902-021-00708-7)
Supplement: Supplementary file 1 — Additional file 1: Supplementary Fig. 1. Correlations between NKX6.1 inactivation level in β cells and Age in non-diabetic subjects. a Correlation between NKX6.1 Nuc-Ins+ cells count and percentage with Aging in Non-diabetic subjects (n = 40). b Correlation between NKX6.1cytIns− cell count and percentage with Aging in non-diabetic subjects (n = 40). Supplementary Fig. 2. Correlations between NKX6.1 inactivation level in β cells and BMI in non-diabetic subjects. a Correlation between NKX6.1 Nuc-Ins+ cells count and percentage with BMI in Non-diabetic subjects (n = 40). b Correlation between NKX6.1cytIns− cell count and percentage with BMI in non-diabetic subjects (n = 40). Supplementary Fig. 3. Correlations between NKX6.1 inactivation level in β cells and HbA1c in non-diabetic subjects. a Correlation between NKX6.1 Nuc-Ins+ cells count and percentage with HbA1c in non-diabetic subjects (n = 40). b Correlation between NKX6.1cytIns− cell count and percentage with HbA1c in non-diabetic subjects (n = 40) [file 12902_2021_708_MOESM1_ESM.docx]

## Supplementary Figures


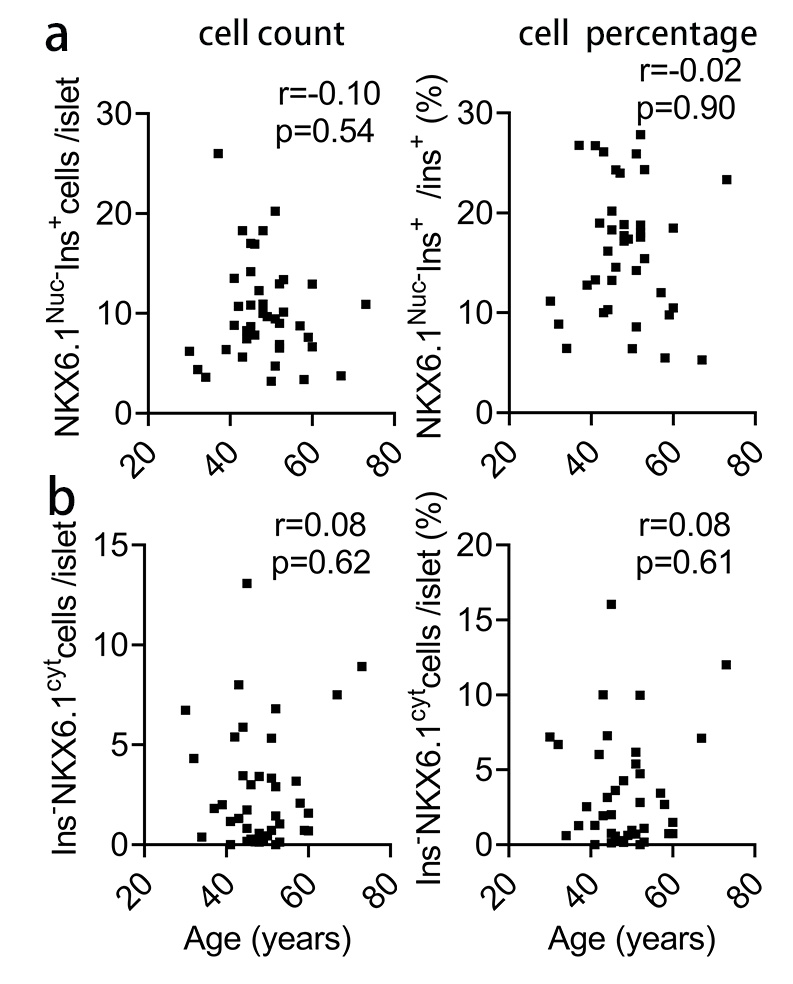


**Supplementary Fig. 1** Correlations between NKX6.1 inactivation level in β cells and Age in non-diabetic subjects. **a** Correlation between NKX6.1 ^Nuc-^Ins^+^ cells count and percentage with Aging in Non-diabetic subjects (n=40). **b** Correlation between NKX6.1^cyt^Ins^-^ cell count and percentage with Aging in non-diabetic subjects (n=40).


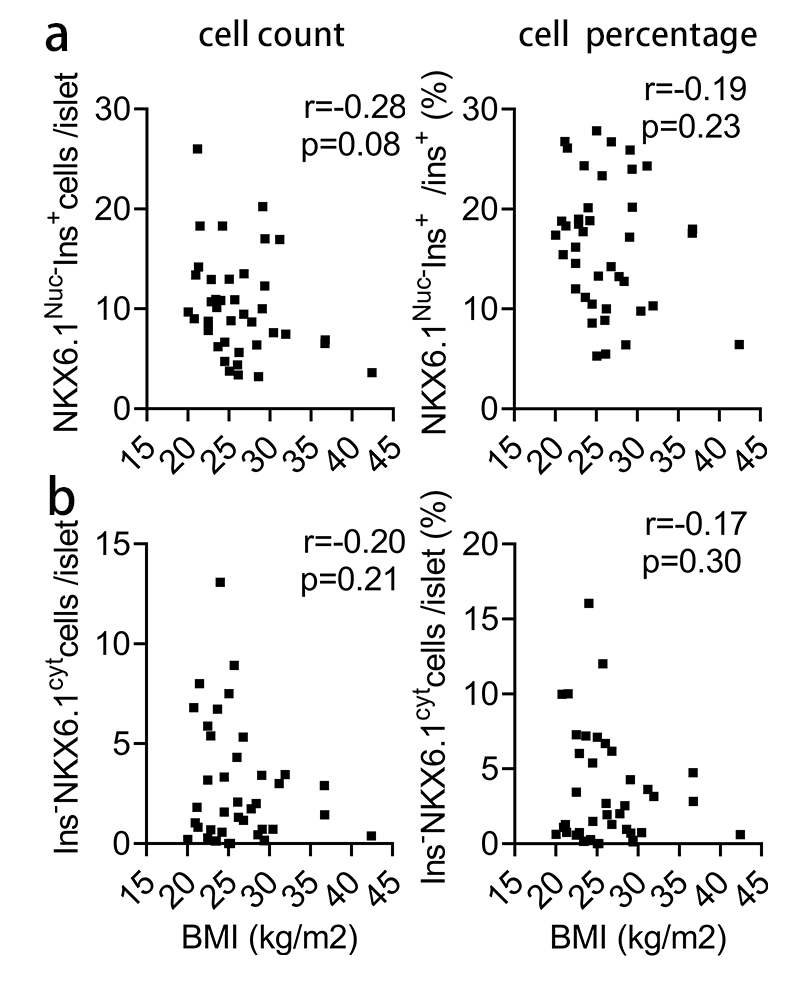


**Supplementary Fig. 2** Correlations between NKX6.1 inactivation level in β cells and BMI in non-diabetic subjects. **a** Correlation between NKX6.1 ^Nuc-^Ins^+^ cells count and percentage with BMI in Non-diabetic subjects (n=40). **b** Correlation between NKX6.1^cyt^Ins^-^ cell count and percentage with BMI in non-diabetic subjects (n=40).


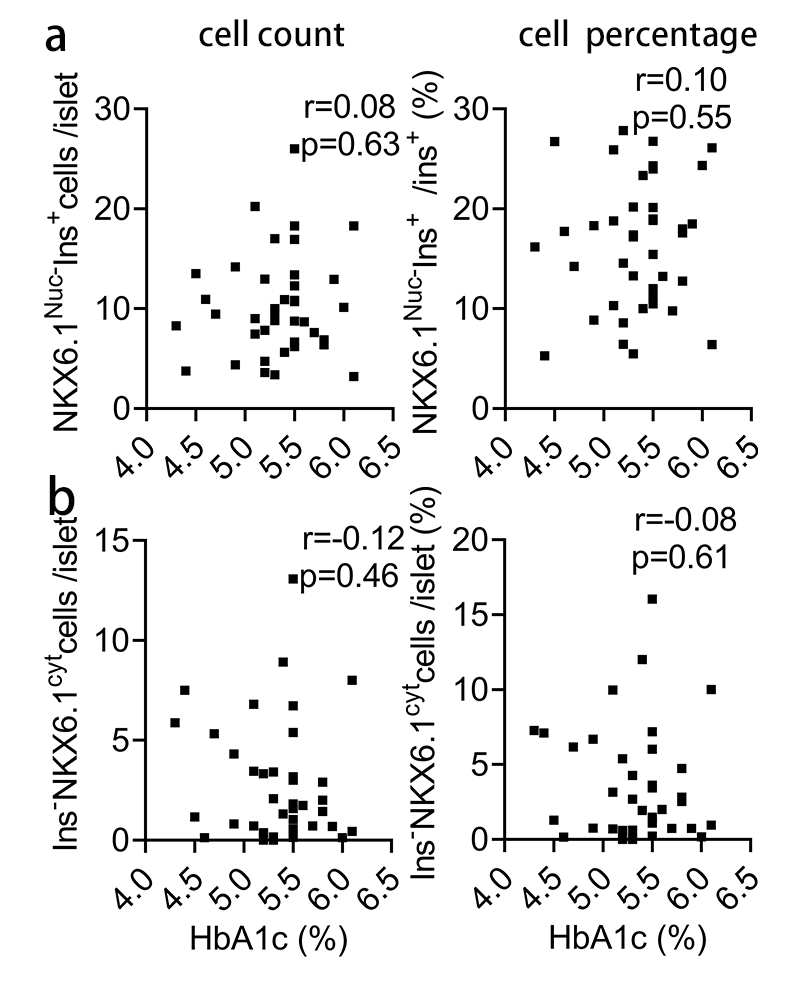


**Supplementary Fig. 3** Correlations between NKX6.1 inactivation level in β cells and HbA1c in non-diabetic subjects. **a** Correlation between NKX6.1 ^Nuc-^Ins^+^ cells count and percentage with HbA1c in non-diabetic subjects (n=40). **b** Correlation between NKX6.1^cyt^Ins^-^ cell count and percentage with HbA1c in non-diabetic subjects (n=40).
